# Processing Coordinate Subject-Verb Agreement in L1 and L2 Greek

**DOI:** 10.3389/fpsyg.2016.00648

**Published:** 2016-05-09

**Authors:** Maria Kaltsa, Ianthi M. Tsimpli, Theodoros Marinis, Melita Stavrou

**Affiliations:** ^1^Language Development Lab, Department of Theoretical and Applied Linguistics, Aristotle University of ThessalonikiThessaloniki, Greece; ^2^Department of Theoretical and Applied Linguistics, University of CambridgeCambridge, UK; ^3^Department of Clinical Language Sciences, School of Psychology and Clinical Language Sciences, University of ReadingReading, UK; ^4^Department of Linguistics, School of Philology, Aristotle University of ThessalonikiThessaloniki, Greece

**Keywords:** number agreement, coordinate subjects, child bilingualism, Greek sentence processing, adult processing

## Abstract

The present study examines the processing of subject-verb (SV) number agreement with coordinate subjects in pre-verbal and post-verbal positions in Greek. Greek is a language with morphological number marked on nominal and verbal elements. Coordinate SV agreement, however, is special in Greek as it is sensitive to the coordinate subject's position: when pre-verbal, the verb is marked for plural while when post-verbal the verb can be in the singular. We conducted two experiments, an acceptability judgment task with adult monolinguals as a pre-study (Experiment 1) and a self-paced reading task as the main study (Experiment 2) in order to obtain acceptance as well as processing data. Forty adult monolingual speakers of Greek participated in Experiment 1 and a hundred and forty one in Experiment 2. Seventy one children participated in Experiment 2: 30 Albanian-Greek sequential bilingual children and 41 Greek monolingual children aged 10–12 years. The adult data in Experiment 1 establish the difference in acceptability between singular VPs in SV and VS constructions reaffirming our hypothesis. Meanwhile, the adult data in Experiment 2 show that plural verbs accelerate processing regardless of subject position. The child online data show that sequential bilingual children have longer reading times (RTs) compared to the age-matched monolingual control group. However, both child groups follow a similar processing pattern in both pre-verbal and post-verbal constructions showing longer RTs immediately after a singular verb when the subject was pre-verbal indicating a grammaticality effect. In the post-verbal coordinate subject sentences, both child groups showed longer RTs on the first subject following the plural verb due to the temporary number mismatch between the verb and the first subject. This effect was resolved in monolingual children but was still present at the end of the sentence for bilingual children indicating difficulties to reanalyze and integrate information. Taken together, these findings demonstrate that (a) 10–12 year-old sequential bilingual children are sensitive to number agreement in SV coordinate constructions parsing sentences in the same way as monolingual children even though their vocabulary abilities are lower than that of age-matched monolingual peers and (b) bilinguals are slower in processing overall.

## Introduction

The present study examines the processing of Subject-Verb (SV) number agreement in pre-verbal and post-verbal coordinate subject constructions in Greek. Examples (1) and (2) illustrate post-verbal and pre-verbal coordinate subject constructions in Greek. Greek has morphological number agreement (singular and plural) marking between the subject and the verb. However, coordinate subjects are a special case because number agreement is sensitive to the position of the subject. In particular, post-verbal coordinate subjects trigger plural agreement but optionally allow for singular verbs as well, as illustrated in example (1) below. In contrast, pre-verbal coordinate subjects require plural agreement while singular number agreement on the verb gives rise to ungrammaticality (Holton et al., 1997; Spyropoulos, 2007; Kazana, 2011), as shown in example (2) below.

1.  Postverbal coordinate subject
       Irthan/Irthe                 o    Yanis ke   i      Maria.
       arrived.3p/arrived.3s  the  Yanis  and  the  Maria
       ‘Yanis and Maria arrived.’
2.  Preverbal coordinate subject
       O    Yanis ke    i      Maria   irthan/^*^irthe.
       the   Yanis  and   the  Maria   arrived.3p/arrived.3s
       ‘Yanis and Maria arrived.’

Agreement has been argued to be either a syntactic (Chomsky, 2001; Bošković, 2009) or an entirely post-syntactic process (Bobaljik, 2008) with Closest Conjunct Agreement (CCA) accounts identifying linear proximity as a key post-syntactic component of grammar (Benmamoun, 1996; Benmamoun et al., 2009; for a detailed analysis on locating agreement see Bhatt and Walkow, 2013). Within syntactic accounts, coordinate subject agreement has been argued to be resolved with either full or partial agreement accounts. In full agreement accounts, agreement takes place with the Coordination Phrase as a whole, while feature mismatch is resolved according to resolution rules (Corbett, 1991). In partial agreement accounts (Aoun et al., 1994), agreement takes place with the closest available conjunct; in post-verbal contexts either with the first or highest conjunct (First Conjunct Agreement, FCA) and in pre-verbal contexts with the last one (Last Conjunct Agreement, LCA). In partial agreement accounts linear order between the coordinated DPs is indirectly addressed within the syntactic component. The phenomenon of partial agreement with coordinate subjects has been attested in many unrelated languages such as Arabic (Aoun et al., 1994), Slovenian (Marušič et al., 2007), Hindi (Benmamoun, 2000), and Serbo-Croatian (Bošković, 2009, 2010). This (mis)match in number agreement patterns may be addressed in two ways; either through VP coordination with verb raising, as in (3), or through DP coordination, as in (4).

3.  [_INFLP_ ν-V  [_νP_ DP_1_ t_ν−V_]   and  [_ν1P_ DP_2_ e_ν−V_]
       arrived        [Yanis arrived] and  [Maria arrived]
4.  [DP [DP_1_ ]and [DP_2_]]

According to Spyropoulos (2007) and Aoun et al. (1994; see also Johannessen, 1998; Harbert and Bahloul, 2002 for similar analyses), number mismatch cases can be accounted for by assuming VP coordination with each conjunct being the subject of its own clause, thus triggering singular agreement there. Verb-raising to the inflection head with deletion of the two lower verb copies results in a surface order whereby the singular verb is followed by two conjoined singular DPs. All other cases, that is, pre-verbal coordinate subjects and post-verbal constructions with plural number agreement, can be accounted for by assuming DP coordination, as in (4). This suggestion is in line with Munn's (1999) phrasal analysis shown to satisfy the requirements for syntactic and semantic plurality when accounting for such agreement phenomena. Notice that the analysis which assumes VP-coordination and verb-raising is syntactically more complex than DP coordination. Specifically, the fact that (3) involves a dependency involving three copies of the verb indicates higher complexity than the structure in (4) where no movement or dependency is formed. In this respect, (4) corresponds more closely to the structure of subject-verb agreement with single, non-coordinate subjects. In addition, plural number agreement with DP coordination (i.e., (4)) is semantically unmarked since the coordinate subject is semantically plural. Finally, plural agreement generalizes over pre-verbal and post-verbal coordinate subjects, whereas the structure in (3) is an option associated with post-verbal coordinate subjects only. This suggests that plural (full) agreement should be more frequent than singular and as such it should be easier to process.

It should also be noted that coordinating a singular and a plural DP subject reduces the acceptability of the singular number option on the verb, as shown by example (5) below. Furthermore, the grammaticality of the coordinate subject with a singular and a plural number DP subject deteriorates further when the plural member precedes the singular one. Compare the examples (5) and (6) below, with one plural and one singular subject DP coordinated:

5.  V + S-**sing** + S-**plural**
       ?Irthe        o   Yanis  ke   ta    pedhia.
       arrived.3s  the Yanis and  the  kids
       ‘Yanis and the kids arrived.’
6.  V + S-**plural** + S-**sing**
       ^*^Irthe        ta   pedhia   ke   o    Yanis.
       arrived.3s  the kids       and  the Yanis
       ‘The kids and Yanis arrived.’

The reduced acceptability of (5) may be reflected in processing patterns and response times too, although such structures have not been investigated in the processing literature yet. The additional effects of the ordering between the singular and the plural subject in (6) increase the variables that number agreement might be sensitive to in coordinate subject processing. Thus, our experimental study examines number agreement in SV and VS constructions with singular DPs only, coordinated as in (1) and (2). Moreover, the possibility of singular subject-verb agreement also appears to be sensitive to other properties of the DPs, such as animacy. In light of Sorace and Keller's (2005) distinctions between hard and soft constraints found in purely syntactic violations vs. syntax-semantics/pragmatics interface violations respectively, Bamyaci et al. (2014) argue that fine-grained distinctions of animacy need be considered for subject-verb agreement in Turkish (for typological observations see Corbett, 2000, 2006; for animacy hierarchy see Haspelmath, 2008). It is not clear whether and how animacy may interact with the Greek subject-verb coordinate agreement structures of the present study. At a first glance, it seems that animate and inanimate DPs allow for singular (partial) agreement with the verb. Consider the example in (7) below:

7.  Xithike     to gala   ke    i     supa.
       spilled.3s  the milk and  the  soup
       ‘The milk and the soup spilled over.’

However, given that inanimate subjects are often “derived” as in unaccusative or passive structures, we did not include animacy as a variable in our study. Instead, the subjects used were, all but one, animate.

Processing studies on subject verb agreement have mainly focused on “attraction” errors in which the verb erroneously agrees with an intervening noun bearing number specification different from the head noun of the subject (Franck et al., 2006; Wagers et al., 2009). Findings suggest that such attraction errors in agreement are attested with ungrammatical sentences and are accounted for by a cue-based retrieval mechanism for accessing and comparing previously processed constituents. Tucker et al. (2015) examined agreement errors within the subject constituent in Arabic and found that morphologically discontinuous plural forms need further elaboration for the grammatical features in order for them to be used as processing cues for the retrieval system. These self-paced reading studies focused on adult monolingual data and it is unclear how and whether these attraction errors would affect learners' (child or adult) processing as well.

Child processing studies on subject-verb agreement violations are limited and do not include coordinate subjects. Brandt-Kobele and Höhle (2014) conducted an eye-tracking study with 3 and 5 year old monolingual German speaking children and found that only the older group was sensitive to (un)grammaticality. Preferential listening studies have reported a high sensitivity of monolingual children as young as 2 years old to subject-verb agreement violations (e.g., Soderstrom et al., 2007; Polišenská, 2010; Nazzi et al., 2011. Nevertheless, no online studies are found in the literature that test coordinate subjects in particular.

In Greek, only a limited number of studies (Spyropoulos, 2007; Kazana, 2011) have addressed the syntactic derivation of such constructions and, primarily, from a theoretical perspective. From the processing perspective, it remains unclear how adults and children process these constructions in real-time, whether they are able to rapidly integrate number information and, show sensitivity to the temporary mismatch between plural number in the verb and singular number on the subject in post-verbal coordinate subjects. Finally, it has not been investigated whether and how monolingual and bilingual speakers of Greek process the (un)grammaticality induced by pre-verbal coordinate subjects and singular number on the verb.

The investigation of coordinate subjects allows us to compare whether children and adults are sensitive to number mismatch between the verb and the subject when processing sentences incrementally, at the point where a mismatch leads to ungrammaticality as soon as the verb is encountered (when the subject is pre-verbal) as opposed to further down in the sentence (when the subject is post-verbal). We anticipate that child data will show the automatic reflex of longer reading times (RTs hereafter) in both cases immediately after the segment in which the mismatch becomes apparent. Sequential bilingual children whose language abilities are usually lower than those of monolingual controls have been shown to be sensitive to SV agreement violations (Chondrogianni and Marinis, 2012) in English sentences with simple subjects despite of their variability in production. The present study investigates whether bilinguals will also be sensitive to subject-verb agreement mismatch in coordinate subjects which are more complex than simple subject DPs.

Finally, given that our bilingual participants are speakers of Albanian and Greek, we considered whether Albanian allows (a) for post-verbal subjects and (b) for partial number agreement with coordinate subjects in post-verbal position. Albanian, like Greek, is a null subject language. As such, post-verbal subjects should be available as a property associated with the null subject parameter (Rizzi, 1986). Although post-verbal subjects are indeed available in Albanian, partial number agreement with post-verbal subjects is accepted by Albanian native speakers but not as strongly as full number agreement (Meniku and Campos, 2016). Moreover, unlike Greek, partial agreement cases are not mentioned in grammar books (Meniku and Campos, 2016). Consider the examples in (8) below (E. Kapia, p.c.):

8.  a. Erdhən         Xhoni  dhe  Maria.
           Arrived.3p   John    and  Maria
       b. Erdhi           Xhoni  dhe  Maria.
           Arrived.3sg. John    and  Maria
           ‘John and Maria arrived.’

Given that L1 and L2 are similar in the relevant respects (post-verbal subjects, full and partial number agreement with post-verbal coordinate subjects), processing data from bilingual Albanian-Greek children should reflect child L2 processing properties rather than (negative) transfer effects.

## Research questions and predictions

Our main research question is how coordinate subjects are processed in terms of subject-verb number agreement in VS and SV constructions in Greek by monolingual and bilingual speakers. To this aim, we developed two experiments, an acceptability judgment task (Experiment 1) and a self-paced reading task as the main study (Experiment 2). Adult monolingual speakers of Greek participated in both experiments so as to establish that the acceptability rates of singular and plural number agreement are indeed sensitive to the position of coordinate subjects (pre-verbal or post-verbal) in the adult grammar and, second, to examine the parsing steps to number resolution. In addition, our study aims to identify whether sequential bilingual children with Albanian as L1 and Greek as L2 process sentences in a way similar to monolingual Greek speaking children in terms of speed and pattern of processing related to SV agreement. This dataset is a valuable addition to the literature of sentence processing in developing grammars (both L1 and L2) and in Greek in particular.

With regard to the adult data, we expect that the availability of partial number agreement in coordinate DPs will be confirmed. In particular, the acceptability data are expected to highlight the difference between pre-verbal and post-verbal coordinate subjects and number agreement options, with post-verbal subjects showing higher tolerance to singular number marking on the verb. Adult processing data are also expected to show sensitivity to the singular-plural number distinction as well as to the singular number option with post-verbal vs. pre-verbal coordinate subjects. However, given the “marked” status of partial agreement discussed above (see Section Introduction), it is possible that adult online data will show a number effect with shorter reading times with plural verbs regardless of subject position.

Turning to the child processing data, we expect that (a) in light of the continuity of parsing hypothesis according to which the structural parser of monolingual children is similar to the adult one (Pinker, 1984; Clahsen and Felser, 2006) monolingual children will show similar processing steps to the adults, and (b) bilingual children will show longer RTs than monolingual children in line with previous sentence processing studies (Marinis, 2007, 2008; Chondrogianni and Marinis, 2012; Chondrogianni et al., 2015). This is partly based on the bilingual children's lower language abilities in their L2 compared to monolingual children. In this study, language ability is measured with an expressive vocabulary test, (see Section Child Participants). In terms of processing patterns for subject-verb number agreement, we expect all participants to show longer RTs in post-verbal subject constructions when the verb is in the plural as opposed to singular because there is a temporary number mismatch between the verb (plural) and the subject (singular) at the first segment following the verb, i.e., the first member of the coordinate subject. In addition, if the derivation which allows singular number marking on the verb with a coordinate subject is different (VP coordination and verb-raising) and more complex than the derivation with plural number marking, we expect a number effect to be attested on the second conjunct or in following segments. If sequential bilingual children process subject-verb agreement qualitatively similarly to monolingual children, the same effect should be attested in both groups of children (for qualitative similarities of bilingual and monolingual children's processing of thematic roles see Marinis and Saddy, 2013). In pre-verbal subject constructions, longer RTs are expected on the verb in singular verb structures compared to plural ones as singular number marking on the verb is ungrammatical in this context. Recall that in pre-verbal structures, coordination is only allowed as DP-coordination leaving plural number as the only agreement option (Aoun et al., 1994; Spyropoulos, 2007).

## Materials and methods

### Experiment 1: acceptability judgment task

#### Participants

Forty adult native speakers of Greek (20 female) were included in Experiment 1. At the time of testing, the mean age was 32 years (age range: 22–60 years old).

#### Experimental design

The acceptability judgment task aimed at testing coordinate subject-verb agreement in Greek manipulating two factors: the subject position (pre-/post-verbal) and the number of the verb (singular/plural). The experiment consisted of 96 items; 24 experimental and 72 filler sentences. The experimental items were of similar syllable length and the DPs were definite, singular and animate (with the exception of one inanimate item); half of the DPs involved proper names. The task was conducted as an online survey that lasted approximately 10–15 min. The participants were instructed to evaluate sentences in Greek in a scale of 1–5 with 1 being the score for an unacceptable sentence in Greek and 5 for a fully acceptable one. The conditions are exemplified in (9–12) below:

9.  Postverbal Subject, V-singular + Subject
       Emfanistike i Maria ke o Yanis meta tin prosklisi.
       appear^-PAST-3SING^  the^-NOM^    Maria^-NOM^   and   the^-NOM^
       Yanis^-NOM^ after the^-ACC^ invitation^-ACC^
10.  Postverbal Subject, V-plural + Subject
         Emfanistikan i Maria ke o Yanis meta tin prosklisi.
         appear^-PAST-3PLUR^   the^-NOM^    Maria^-NOM^   and   the^-NOM^
         Yanis^-NOM^ after the^-ACC^ invitation^-ACC^
11.  Preverbal Subject, Subject + V-singular
         ^*^I Maria ke o Yanis emfanistike meta tin prosklisi.
         the^-NOM^       Maria^-NOM^      and       the^-NOM^     Yanis^-NOM^
         appear^-PAST-3SING^ after the^-ACC^ invitation^-ACC^
12.  Preverbal Subject, Subject + V-plural
         I Maria ke o Yanis emfanistikan meta tin prosklisi.
         the^-NOM^       Maria^-NOM^     and        the^-NOM^      Yanis^-NOM^
         appear^-PAST-3PLUR^ after the^-ACC^ invitation^-ACC^
         ‘Maria and John turned up after the invitation.’

Out of the 72 filler sentences, half of them were well-formed grammatical sentences (N: 36), as in (5) below, and half ungrammatical (N: 36) as in (13–14) below. Ungrammaticality was always due to violations of inflectional features, such as gender, number or case.

13.  I vivliothiki sti sofita ehi pola leromena rafia.
         the^-NOM^       bookcase^-NOM^        in-the^-ACC^         attic^-ACC^
         have^-PRES-3SING^ plenty^-ACC^ dirty^-ACC^ shelves^-ACC^
         ‘The bookcase in the attic has a lot dirty shelves.’
14.  ^*^I proti katiki tis neas ipirou efaye rizes
         the^-NOM^   first^-NOM^    inhabitants^-NOM^   the^-GEN^    new^-GEN^
         continent^-GEN^ eat^-PAST-3SING^ roots^-ACC^
         ‘The first inhabitants of the new continent ate roots.’

The Experimental materials were divided into 4 lists in a Latin Square design and fillers were identical in all lists.

#### Results

**Figure 1:**
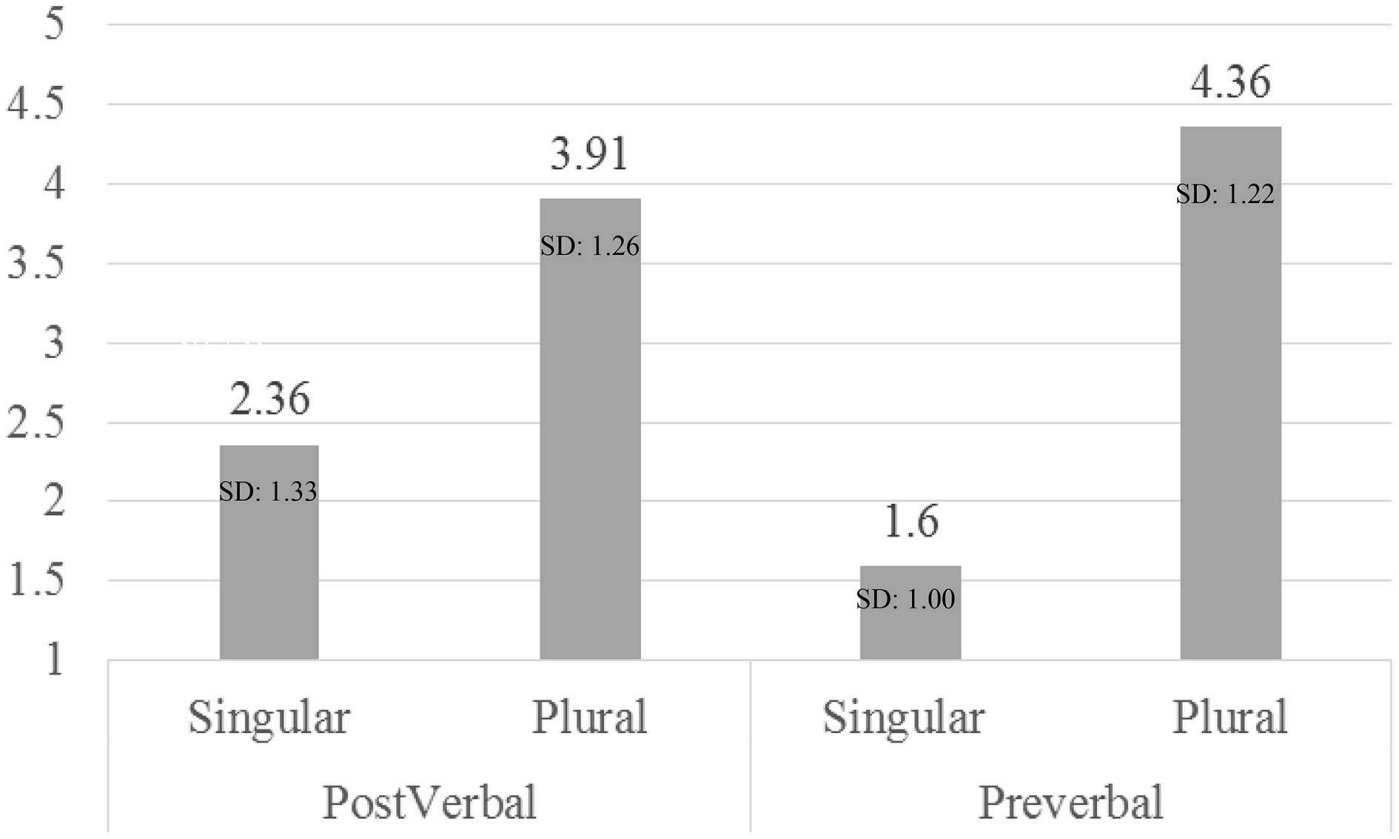
**Acceptability judgment task—number and subject position**.

To analyze the acceptability data we performed repeated measures analysis of variance (ANOVA) with Number (singular vs. plural) and Subject Position (pre-verbal, post-verbal) as the within subjects variables. Figure 1 shows the results of the acceptability judgment.

The analysis showed a main effect of Number [*F*_(1, 239)_ = 571.069, *p* < 0.001, $\eta_{p}^{2}$ = 0.705], a main effect of Subject Position [*F*_(1, 239)_ = 6.052, *p* = 0.015, $\eta_{p}^{2}$ = 0.025], and an interaction between Number and Subject Position [*F*_(1, 239)_ = 92.518, *p* < 0.001, $\eta_{p}^{2}$ = 0.279; see Figure 1]. Within both types of structures plural receives higher acceptability scores than singular [Post-verbal: *F*_(1, 239)_ = 197.037, *p* < 0.001, $\eta_{p}^{2}$ = 0.452; Pre-verbal: *F*_(1, 239)_ = 639.387, *p* < 0.001, $\eta_{p}^{2}$ = 0.728]. However, when examining the differences between the two types of structures the comparisons show that singular is more acceptable with post-verbal subjects [*F*_(1, 239)_ = 72.419, *p* < 0.001, $\eta_{p}^{2}$ = 0.233], while plural with pre-verbal ones [*F*_(1, 239)_ = 26.556, *p* < 0.001, $\eta_{p}^{2}$ = 0.100].

The results of the acceptability judgment task establish the difference in the acceptance rates of singular VPs when their subject precedes or follows them. The online study will identify the processing steps building up the interpretation of those constructions in the adult and child data.

### Experiment 2: self-paced reading task

#### Adult participants

One hundred and forty one adult native speakers of Greek (102 female) were included in the main study. At the time of testing, the mean age was 24 years (age range: 18–59 years old). None of those participants completed the acceptability judgment task.

#### Child participants

Thirty Greek-Albanian sequential bilingual children (11 female) and forty one monolingual Greek children (33 girls) participated in this study. At the time of testing, the mean age of the bilingual group was 11;3 (age range: 10;3–12;7, standard deviation (SD): 0;6) and the mean age of the monolingual group was 11;2 years of age (age range: 10;2–12;2, SD: 0;5). There was no significant difference in age between the two groups [*F*_(1, 69)_ = 0.101, *p* = 0.752, $\eta_{p}^{2}$ = 0.062]. All participants in the study were typically developing without any history of speech and/or language disorder.

All participants attend monolingual state schools where Greek is used as the majority language. To assess the language history and homogeneity of our bilingual group we collected information on our participants' home language practices in preschool years, early (preschool) and current (bi-)literacy skills, and current language preferences for speaking and listening in daily communication, through extensive questionnaires. Specifically, *home language* questions referred to the child's exposure to each and to both languages from birth up to the age of schooling, i.e., around age 6. The *early (bi)literacy* questions asked for information about whether and in which language(s) family members read books to the child. Questions on *current (bi)literacy* asked for information about children's current language preference/use in writing/reading tasks, and, more specifically, (a) whether the children took language classes in Albanian (L1) and (b) which language was their preferred one for daily writing/ reading tasks (writing lists/letters/cards, reading aloud, texting, emailing, visiting websites, video-gaming, book/magazine reading). Finally, the *current language use* questions asked for the child's language preference/use in oral tasks such as the child's preferred language for oral interaction with family members/friends, for memorizing phone numbers, telling the time, mental counting/calculating and for watching TV/movies. Their answers were used to generate four composite input scores for (a) Greek, (b) Albanian and (c) both languages options.

The children's lexical abilities in Greek were assessed in both languages. A standardized expressive vocabulary test was used for Greek (Vogindroukas et al., 2009, adaptation from Renfrew) and an adaptation of the same task was used for Albanian (Kapia and Kananaj, 2013). These tests provided us with independent measures of our participants' language proficiency in their L1 and L2. To examine whether our bilingual participants formed a homogenous group we examined the factorability of the input factors extracted from the questionnaires and their vocabulary development in each language.

The factorability of all input factors was examined to determine the personal characteristics of the bilingual participants that might further influence their responses. A Principal Axis Factor (PAF) with a Varimax (orthogonal) rotation was conducted on the bilinguals' input profiles. The Kaiser-Meyer-Olkin measure of sampling adequacy was 0.56, close to the recommended value of 0.6, and Bartlett's test of sphericity was significant [$\chi_{\left(136\right)}^{2}$ = 779.129, *p* < 0.001] and only loadings >0.30 were considered relevant. The factor analysis showed that 30% of the variance of the data set is explained by the development of Greek lexical abilities and 23% of the variance by the Albanian vocabulary development. Both Greek and Albanian vocabulary scores were very close to normally distributed (see Table 1). Out of the questionnaire questions only the home language practices appear to explain some of the variance, with Greek-dominant home practices explaining 9%, Albanian-dominant 9%, and bilingual home ones 8% of the total variance.

**Table 1:** **Descriptive statistics for the factorable bilingual profile characteristics (N: 30)**.

	**Mean (SD)**	**Skewness (Std. Error)**	**Kurtosis (Std. Error)**
Greek Vocabulary	73% (14)	−0.86 (0.42)	0.07 (0.83)
Albanian Vocabulary	65% (18)	−0.23 (0.42)	−0.59 (0.83)
Greek-dominant Home	31% (18)	0.85 (0.42)	1.08 (0.83)
Albanian-dominant Home	37% (22)	0.49 (0.42)	0.45 (0.83)
Bilingual Home	31% (19)	−0.003 (42)	−0.96 (0.83)

Given the outcome of the factor analysis with respect to the role of vocabulary skills in each language and given that the experimental study is a reading task in Greek, we divided the children in two groups; those who scored higher than the mean (+1SD) in the Greek vocabulary task (Group A hereafter, N: 19) and those who scored lower that the mean (Group B hereafter, N: 11). The two groups will be considered in relation to their performance on the self-paced reading task. It is noteworthy that the bilinguals' scores on Greek vocabulary is equivalent to that of 8-year-old monolingual children, indicating at least a 2-year gap in lexical development compared to monolingual controls.

#### Experimental design

A self-paced reading task^1^ was used to investigate how participants process coordinate subject-verb agreement in Greek. The task manipulated two factors: Subject Position (pre-/post-verbal) and Number marking on the verb (singular/plural). The experiment consisted of 106 items; 10 practice sentences, 24 experimental sentences and 72 filler sentences. All experimental and filler items were identical to those of Experiment 1 (Acceptability Judgment Task). Participants controlled the speed of reading each segment by pressing a button on the keyboard. The button press recorded the participants' reading times (RT) per segment. Sentences were segmented in six reading areas: the “Verb,” “Subject,” “And,” “Subject,” “PP”/”AdvP” (split in two segments) as in (15–18) below. Slashes indicate segments. Each segment appeared in the middle of the screen and was replaced by the following segment after the participant pressed the spacebar. The last segment appeared with a full stop.

15.  V-singular + Postverbal Subject
         Emfanistike / i Maria / ke / o Yanis / meta / tin prosklisi.
         appear^-PAST-3SING^ / the^-NOM^ Maria^-NOM^ / and / the^-NOM^
         Yanis^-NOM^ / after / the^-ACC^ invitation^-ACC^
16.  V-plural + Postverbal Subject
         Emfanistikan / i Maria / ke / o Yanis / meta / tin prosklisi.
         appear^-PAST-3PLUR^ / the^-NOM^ Maria^-NOM^ / and / the^-NOM^
         Yanis^-NOM^ / after / the^-ACC^ invitation^-ACC^
17.  Preverbal Subject + + V-singular
         ^*^I Maria / ke / o Yanis / emfanistike / meta / tin prosklisi.
         the^-NOM^   Maria^-NOM^    /   and   /   the^-NOM^  Yanis^-NOM^   /
         appear^-PAST-3SING^ / after / the^-ACC^ invitation^-ACC^
18.  Preverbal Subject + V-plural
         I Maria / ke / o Yanis / emfanistikan / meta / tin prosklisi.
         the^-NOM^    Maria^-NOM^   /   and   /   the^-NOM^    Yanis^-NOM^   /
         appear^-PAST-3PLUR^ / after / the^-ACC^ invitation^-ACC^
         ‘Maria and John turned up after the invitation.’

As with Experiment 1, out of the 72 filler sentences half were well-formed (N: 36) as in (19) and half ungrammatical (N: 36) as in (20) below. Ungrammaticality in filler items was due to inflectional features such as gender, number or case. Segments are presented in (19) and (20):

19.  I vivliothiki / sti sofita / ehi / pola / leromena / rafia.
         the^-NOM^       bookcase^-NOM^        in-the^-ACC^         attic^-ACC^
         have^-PRES-3SING^ plenty^-ACC^ dirty^-ACC^ shelves^-ACC^
         ‘The bookcase in the attic has a lot dirty shelves.’
20.  ^*^I proti / katiki / tis neas / ipirou / efaye / rizes.
         the^-NOM^    first^-NOM^   inhabitants^-NOM^   the^-GEN^    new^-GEN^
         continent^-GEN^ eat^-PAST-3SING^ roots^-ACC^
         ‘The first inhabitants of the new continent ate roots.’

Yes-no comprehension questions were included for 30% of the total number of sentences to ensure that participants were reading for comprehension. Each question appeared on the screen and participants had to indicate whether the answer was “yes” or “no” by pressing one of the two pre-specified buttons on the keyboard. As with Experiment 1, four lists were created using a Latin Square design. Fillers were identical in all lists. The experiment lasted approximately 15 min.

#### Results: adult data

The responses on the comprehension questions were used to ensure that participants attended to the content of the sentences. A minimum of 90% accuracy on the comprehension questions established that participants were attending and no participant had to be eliminated from further analysis. The variables considered were number on the verb and subject position, i.e., whether the subject appeared pre-verbally or post-verbally. The data obtained included reading times (RTs) on each segment. RTs were screened for extreme values and outliers. Outliers were defined as RTs above or below 2 standard deviations from the mean RT in each condition separately per subject and item. Outliers were replaced with the mean RT for each condition per subject and item once this value was removed. Extreme values and outliers comprised 2.7% of the adult data (564 out of 20304 data points). Post-verbal and pre-verbal structures were analyzed separately because segments included different words due to the word-order difference. In each data set (post-verbal and pre-verbal structures) we performed repeated measures analysis of variance (ANOVA) with Number (singular vs. plural) as the within subjects factor.

The analysis of post-verbal structures (Table 2) showed a main effect of number on the 2nd, 5^th^, and 6th segments. Specifically, the per subject analysis showed that the participants processed the segment immediately after the verb significantly faster in the singular compared to the plural condition [2nd Segment: *F*_1(1, 140)_ = 4.992, *p* = 0.027, $\eta_{p}^{2}$ = 0.035] but the singular condition was processed significantly slower than the plural condition the last two sentential segments [5th Segment: *F*_1(1, 140)_ = 5.477, *p* = 0.021, $\eta_{p}^{2}$ = 0.038; 6th Segment: *F*_1(1, 140)_ = 14.566, *p* < 0.001, $\eta_{p}^{2}$ = 0.095]. The per item analysis verified the number effect only on the final segment with the plural condition being processed significantly faster than the singular condition [*F*_2(1, 23)_ = 6.819, *p* = 0.016, $\eta_{p}^{2}$ = 0.229].

**Table 2:** **Adult reading times (in milliseconds) per segment in the postverbal subject condition (SDs in parentheses)**.

**Segments**	**1st verb**	**2nd subject**	**3rd conjunct**	**4th subject**	**5th other**	**6th other**
Verb singular	722 (321)	554 (193)	478 (145)	552 (200)	514 (166)	698 (353)
Verb plural	734 (318)	568 (201)	473 (131)	551 (211)	500 (146)	646 (291)

The analysis of pre-verbal structures (Table 3) showed a main effect of Number only on the 5th segment with shorter RTs in the plural compared to the singular condition, similarly to the post-verbal findings [*F*_1(1, 140)_ = 18.924, *p* < 0.001, $\eta_{p}^{2}$ = 0.120; *F*_2(1, 23)_ = 6.955, *p* = 0.015, $\eta_{p}^{2}$ = 0.232].

**Table 3:** **Adult reading times (in milliseconds) per segment in preverbal subject condition (SDs in parentheses)**.

**Segments**	**1st subject**	**2nd coordinate**	**3rd subject**	**4th verb**	**5th other**	**6th other**
Verb singular	712 (290)	467 (136)	511 (177)	560 (207)	525 (156)	675 (306)
Verb plural	712 (294)	476 (138)	526 (187)	565 (210)	502 (139)	666 (321)

#### Results: child data

Responses on the comprehension questions were used to ensure participants' attention; both bilingual and monolingual children had a minimum of 80% accuracy in those questions and thus no participant was eliminated from further analysis. As with the adult data, the variable examined was Number on the verb with coordinate subjects appearing either post-verbally or pre-verbally. The data obtained included RTs on each segment. RTs were screened for extreme values and outliers. Extreme values (over 10 s) were identified for each condition separately per subject and item and were removed, leading to the removal of four instances. Extreme values and outliers comprised 0.7% of the bilingual data (29 out of 4320 data points) and 0.9% of the monolingual data (56 out of 5904 data points). In each structure we performed repeated measures analysis of variance (ANOVA) with Number (singular vs. plural) as the within subjects factor and Group (bilinguals vs. monolinguals) as the between subjects factor.

The analysis of post-verbal structures (Table 4) revealed a main effect of Group across all segments suggesting overall longer RTs in bilinguals compared to monolingual children [1st Segment: *F*_1(1, 70)_ = 13.951, *p* < 0.001, $\eta_{p}^{2}$ = 0.168; *F*_2(1, 47)_ = 21.610, *p* < 0.001, $\eta_{p}^{2}$ = 0.320; 2nd Segment: *F*_1(1, 70)_ = 22.996, *p* < 0.001, $\eta_{p}^{2}$ = 0.250; *F*_2(1, 47)_ = 52.276, *p* < 0.001, $\eta_{p}^{2}$ = 0.532; 3rd Segment: *F*_1(1, 70)_ = 13.802, *p* < 0.001, $\eta_{p}^{2}$ = 0.167; *F*_2(1, 47)_ = 37.937, *p* < 0.001, $\eta_{p}^{2}$ = 0.452; 4th Segment: *F*_1(1, 70)_ = 16.254, *p* < 0.001, $\eta_{p}^{2}$ = 0.191; *F*_2(1, 47)_ = 24.536, *p* < 0.001, $\eta_{p}^{2}$ = 0.348; 5th Segment: *F*_1(1, 70)_ = 15.973, *p* < 0.001, $\eta_{p}^{2}$ = 0.188; *F*_2(1, 47)_ = 30.448, *p* < 0.001, $\eta_{p}^{2}$ = 0.398; 6th Segment: *F*_1(1, 69)_ = 17.479, *p* < 0.001, $\eta_{p}^{2}$ = 0.202; *F*_2(1, 47)_ = 18.984, *p* < 0.001, $\eta_{p}^{2}$ = 0.291]. Moreover, a main effect of Number on Segment 2, i.e., the first DP immediately after the verb, was found. Specifically, RTs on the first DP were significantly longer when the verb was in the plural than in the singular [2nd Segment: *F*_1(1, 70)_ = 13.729, *p* < 0.001, $\eta_{p}^{2}$ = 0.166; *F*_2(1, 46)_ = 13.055, *p* < 0.001, $\eta_{p}^{2}$ = 0.321]. An interaction of Group by Number was only found on the last segment [6th Segment: *F*_1(1, 70)_ = 4.402, *p* = 0.040, $\eta_{p}^{2}$ = 0.060; *F*_2(1, 46)_ = 4.124, *p* = 0.048, $\eta_{p}^{2}$ = 0.082] with bilingual children showing longer RTs in plural compared to the singular VPs (*p* < 0.001) and monolingual children longer RTs with singular compared to plural VPs (*p* < 0.001).

**Table 4:** **Child reading times (in milliseconds) per segment in postverbal subject condition (SDs in parentheses)**.

**Segments**		**1st verb**	**2nd subject**	**3rd conjunct**	**4th subject**	**5th other**	**6th other**
Bilinguals	Singular	1543 (1082)	1237 (690)	823 (360)	1281 (926)	917 (509)	1104 (616)
	Plural	1455 (830)	1360 (707)	845 (343)	1306 (795)	898 (396)	1178 (704)
Monolinguals	Singular	1087 (627)	831 (407)	653 (229)	879 (602)	708 (265)	850 (459)
	Plural	1100 (595)	942 (474)	688 (300)	874 (472)	705 (234)	799 (371)

In the pre-verbal subject condition (Table 5), a main effect of Group across all segments was also found due to the longer RTs in bilingual compared to monolingual children [1st Segment: *F*_1(1, 70)_ = 10.207, *p* = 0.002, $\eta_{p}^{2}$ = 0.129; *F*_2(1, 47)_ = 12.883, *p* < 0.001, $\eta_{p}^{2}$ = 0.219; 2nd Segment: *F*_1(1, 70)_ = 11.607, *p* = 0.001, $\eta_{p}^{2}$ = 0.144; *F*_2(1, 47)_ = 34.516, *p* < 0.001, $\eta_{p}^{2}$ = 0.429; 3rd Segment: *F*_1(1, 70)_ = 17.160, *p* < 0.001, $\eta_{p}^{2}$ = 0.199; *F*_2(1, 47)_ = 24.691, *p* < 0.001, $\eta_{p}^{2}$ = 0.349; 4th Segment: *F*_1(1, 70)_ = 12.636, *p* = 0.001, $\eta_{p}^{2}$ = 0.155; *F*_2(1, 47)_ = 24.794, *p* < 0.001, $\eta_{p}^{2}$ = 0.350; 5th Segment: *F*_1(1, 70)_ = 9.256, *p* = 0.003, $\eta_{p}^{2}$ = 0.118; *F*_2(1, 47)_ = 15.728, *p* < 0.001, $\eta_{p}^{2}$ = 0.255; 6th Segment: *F*_1(1, 70)_ = 15.694, *p* < 0.001, $\eta_{p}^{2}$ = 0.185; *F*_2(1, 47)_ = 16.365, *p* < 0.001, $\eta_{p}^{2}$ = 0.262]. Moreover, a main effect of Number on the segment immediately after the verb was revealed: longer RTs were found after singular verbs compared to RTs for segments following plural verbs [5th Segment: *F*_1(1, 70)_ = 7.051, *p* = 0.010, $\eta_{p}^{2}$ = 0.093; *F*_2(1, 47)_ = 7.720, *p* = 0.008, $\eta_{p}^{2}$ = 0.144]. Lastly, no interaction of Group by Number was found suggesting that bilingual and monolingual children process pre-verbal structures similarly.

**Table 5:** **Child reading times (in milliseconds) per segment in preverbal subject condition (SDs in parentheses)**.

**Segments**		**1st subject**	**2nd conjunct**	**3rd subject**	**4th verb**	**5th other**	**6th other**
Bilinguals	Singular	1476 (1083)	830 (641)	1246 (721)	1201 (679)	868 (343)	1197 (703)
	Plural	1349 (767)	794 (323)	1262 (991)	1234 (669)	804 (293)	1096 (626)
Monolinguals	Singular	1095 (862)	663 (247)	817 (410)	895 (484)	722 (263)	850 (438)
	Plural	1040 (530)	660 (209)	872 (521)	928 (470)	696 (225)	846 (408)

Lastly, we tested the interaction of the key factorable characteristic of our bilinguals namely Greek vocabulary scores [Group A (high) vs. Group B (low)], with Group as the between subjects factor and Number as the within subjects factor. Both in the post-verbal and pre-verbal conditions no interaction was detected (*p* > 0.05), suggesting that their vocabulary skills did not affect their syntactic processing of coordinate subjects.

## Discussion

The present study examined the processing of Number agreement between coordinate subjects consisting of two singular DPs and the verb, in sentences with the coordinate subject being either in pre-verbal or in post-verbal position. The language studied is Greek and the data included monolingual children and adult Greek speakers and sequential bilingual Albanian-Greek children. Since subject verb agreement is sensitive to both hierarchical and linear (adjacency) constraints, the online data would shed light in the relationship between grammar and parser. Specifically, Greek presents a special case for coordinate subject-verb agreement. Verbs are marked for singular and plural number. Number agreement with coordinate subjects is sensitive to the position of the coordinate subject in that while plural number is the only option with pre-verbal subjects, singular is also possible when the coordinate subject is post-verbal (Spyropoulos, 2007; Kazana, 2011). This is an instance of “partial” agreement attested in other languages too (for Arabic see Aoun et al., 1994, for Slovenian see Marušič et al., 2007, for Hindi see Benmamoun, 2000, and for Serbo-Croatian see Bošković, 2009, 2010). In order to confirm that the singular is indeed an acceptable option in adult Greek we presented an acceptability judgment task including all the sentences used in the online self-paced reading task to a group of adult native speakers of Greek. The results confirmed our predictions. Specifically, singular number agreement in sentences with post-verbal coordinate subjects was significantly more acceptable than in sentences with pre-verbal coordinate subjects. Plural agreement on the other hand was acceptable regardless of subject position.

As suggested in Section Introduction, the derivation of partial agreement (singular verb) involves VP-coordination and V-raising (Aoun et al., 1994; Munn, 1999; Spyropoulos, 2007). In contrast, full agreement assumes DP-coordination and no movement dependency formed. In terms of processing cost, we thus expect that partial agreement would be more complex than full agreement not only because the derivation requires more steps but also because full agreement maps directly onto semantic number agreement while partial agreement does not. In addition, partial agreement is only available with post-verbal coordinate subjects while full agreement is available in all contexts. This restriction adds to the markedness of partial agreement and the associated increased complexity. On these grounds, we predicted that plural agreement would be preferred in online processing showing a number effect at least in the last segments of the sentence with both pre-verbal and post-verbal coordinate subjects. The preference for plural number agreement in both contexts was expected to be found in all groups, although adults were expected to be faster than children, and monolingual children faster than bilinguals. Bilingual children were also expected to show a stronger number effect in the post-verbal condition than monolingual children given the more marginal status of partial agreement with coordinate post-verbal subjects in Albanian (Meniku and Campos, 2016). A number effect was also expected to be found in all groups in the first DP appearing after the plural verb in the post-verbal coordinate subject condition given the local number mismatch. Finally, in the case of pre-verbal subject structures, delays were expected on the singular verb since the coordinate subjects have already been presented and ungrammaticality would be detected on the singular verb itself. This effect should be visible in the performance of both monolingual and bilingual children as well as in monolingual adult data.

Our results showed that the overall sentence processing patterns of all groups was similar with number effects being found in all groups in a similar way: plural number was processed faster in final segments than singular in both the pre-verbal and the post-verbal subject condition. The monolingual child data appear to support the continuity of parsing hypothesis (Pinker, 1984; Clahsen and Felser, 2006) since the parsing of monolingual children was similar to the adult one. Differences were found in terms of speed of processing; bilingual children were significantly slower compared to monolingual children. In the pre-verbal condition, monolingual and bilingual children performed similarly showing a number effect indicating that they detected ungrammaticality. The data showed a number effect both in the post-verbal and pre-verbal conditions on the segments following the verb; specifically, in post-verbal constructions there was a main effect of number on the first coordinated subject immediately after the verb with plural verbs delaying significantly the processing, and in pre-verbal constructions on the segment following the verb with singular significantly delaying the processing. As anticipated, monolingual and bilingual children did not differ from each other in the pre-verbal condition but we did find an interaction of group by number on the last sentential segment in the post-verbal condition with bilingual children showing slower processing with plural VPs and monolingual children with singular VPs. Monolingual children showed a number effect with faster processing for plural verb structures with both pre-verbal and post-verbal coordinate subjects. We take this effect to indicate that for monolingual children, DP-coordination is used for coordinate subject processing regardless of the subject position. On the other hand, we interpret bilingual children's slower processing of plural verb structures with post-verbal coordinate subjects as an indication of a reanalysis difficulty. In this condition, they encounter a (plural) verb as the first segment of the sentence followed by a singular DP that would be ungrammatical if this was the subject of the sentence. At this point, both monolingual and bilingual children show longer RTs in this condition compared to the condition in which the verb and the first DP are in the singular. The difference between the two groups is however found at the end of the sentence. Monolingual children show shorter RTs in plural compared to singular conditions, a pattern that is similar to adults and demonstrates that they have integrated the two DPs in the coordinated subject construction into a single subject DP and have matched the plurality of the subject with the verb in the plural. Therefore, the condition with a verb in the singular shows elevated RTs. The bilingual children, on the other hand, still have elevated RTs for the condition, in which there was an initial mismatch in number between the plural verb and the first DP in the singular. This could be argued to indicate a difficulty in the reanalysis of their initial parse (first DP is the subject) and integrate the two coordinated DPs as the subject of the verb (for similar effects on the processing of passives see Marinis and Saddy, 2013). Finally, in the pre-verbal subject condition that tests grammaticality, the number effect is found in both groups in the expected direction.

In conclusion, our study supports findings from other online studies (Marinis, 2007, 2008; Chondrogianni and Marinis, 2012; Chondrogianni et al., 2015) suggesting that bilingual children are slower in incremental processing but not qualitatively different from monolingual children in the grammaticality condition. In this respect, our findings showing a similar number effect in pre-verbal coordinate subjects in bilingual and monolingual children suggest that (un)grammaticality is detected in a similar fashion by the two groups. In contrast, post-verbal structures with coordinate subjects are similar in the two groups only with respect to the delay effect on the first conjunct after the plural verb. This demonstrates again that both groups are sensitive to grammaticality effects in subject-verb agreement constructions. In the final segment, the fact that the two groups show a number effect in the opposite direction is interpreted as a reanalysis and integration problem shown by bilingual children only. Monolingual children show similar processing preferences for plural verbs with coordinate subjects regardless of the subject position. This finding could be interpreted as a VP-coordination and V-raising option being more costly and further delayed in development than the DP-coordination option. Further research into online processing and acceptability judgments of coordinate subjects involving singular and plural DPs as well as pronoun coordination in pre- and post-verbal subject position is required to shed light on the status of the two coordination options.

## Author contributions

MK contributed 40% with the setup of the experiment, the data collection and the data analysis. IT contributed 20% as the PI of the research project in which this research is embedded and with the theoretical contribution to the phenomenon investigated, the design of the experiment and the interpretation of the data. TM contributed to the data presentation and write-up and the interpretation of the data. MS contributed to the design of the critical sentences and the theoretical background of the research question.

### Conflict of interest statement

The authors declare that the research was conducted in the absence of any commercial or financial relationships that could be construed as a potential conflict of interest.

## Footnotes

^1^Stimuli were presented using E-Prime 2.0 software (Psychology Software Tools, 2012. http://www.pstnet.com.).
